# Invasive bacterial disease in young infants in rural Gambia: Population-based surveillance

**DOI:** 10.7189/jogh.13.04106

**Published:** 2023-09-29

**Authors:** Yekini A Olatunji, Adeshola A Banjo, Alexander Jarde, Rasheed Salaudeen, Malick Ndiaye, Lobga B Galega, Aderonke Odutola, Ilias M Hossain, Chidiebere Osuorah, Muhammad S Sahito, Bilquees Shah Muhammad, Nurudeen U Ikumapayi, Momodou M Drammeh, Ahmad Manjang, Richard A Adegbola, Brian M Greenwood, Philip C Hill, Mackenzie A Grant

**Affiliations:** 1 Medical Research Council Unit, The Gambia at London School of Hygiene and Tropical Medicine, Fajara Banjul, The Gambia; 2 Nigerian Institute of Medical Research, Lagos, Nigeria; 3 London School of Hygiene & Tropical Medicine, Department of Disease Control, London, England, UK; 4 Centre for International Health, University of Otago, Dunedin, New Zealand; 5 Murdoch Children’s Research Institute, Melbourne, Australia; 6 Department of Paediatrics, University of Melbourne, Melbourne, Australia

## Abstract

**Background:**

Invasive bacterial diseases (IBD) cause significant mortality in young infants. There are limited population-based data on IBD in young infants in Sub-Saharan Africa.

**Methods:**

We conducted population-based surveillance for IBD among infants aged 0-90 days in a demographic surveillance area in rural Gambia between 1 March 2011 and 31 December 2017. Infants admitted to health facilities within the study area had standardised clinical evaluation plus conventional microbiological investigation. We defined IBD as isolation of pathogenic bacteria from blood, cerebrospinal fluid, lung, or pleural aspirate. We determined incidence, aetiology and case-fatality of IBD.

**Results:**

A total of 3794 infants were admitted and 3605 (95%) had at least one sample collected for culture. We detected 254 (8.0%) episodes of IBD (bacteraemia 241; meningitis 14; pneumonia seven). The incidence of IBD in infants aged 0-90 days was 25 episodes/1000 person-years (95% confidence interval (CI) = 22-28), the incidence in neonates was 50 episodes/1000 person-years (95% CI = 43-58) and the incidence in infants aged 29-90 days was 12 episodes/1000 person-years (95% CI = 9-15). The most common pathogens causing IBD were *Staphylococcus aureus* (n = 102, 40%), *Escherichia coli* (n = 37, 15%), *Streptococcus pneumoniae* (n = 24, 9%) and *Klebsiella pneumoniae* (n = 12, 5%). Case-fatality was 29% (95% CI = 23-37) in neonates and 19% (95% CI = 11-29) in infants aged 29-90 days. A minimum of 7.3% of all young infant deaths in the population were caused by IBD.

**Conclusions:**

IBD are common in young infants in rural Gambia and have a high case-fatality. Strategies are needed to prevent IBD in young infants. Overcoming barriers to widespread implementation of existing vaccines and developing new vaccines against the most common pathogens causing IBD should be among top priorities for reducing the high mortality rate in young infants.

Despite the recent global decline in under-5 mortality, mortality in young infants, particularly neonates, remains high in most low-resource countries. Globally, an estimated 5.0 million children under the age of five years died in 2020, of whom 2.4 million (48%) were in the first month of life [1]. Sub-Saharan Africa accounts for over half (54%) of this mortality, with 2.7 million estimated annual under-5 deaths [1]. According to the MICS 2018 survey, infant mortality in The Gambia is 41 per 1000 live births and neonatal mortality is 25 per 1000 live births [1]. Serious bacterial infections account for about 44% of neonatal admissions [2]. Beyond the first month of life, most other infant deaths occur during the second and third months of life [3] and infectious diseases remain a leading cause of these deaths [4]. Invasive bacterial diseases (IBD), such as sepsis, meningitis, and pneumonia are estimated to cause about a third of the 2.5 million annual neonatal deaths globally [5]. In 2019, 7.7 million deaths were linked to bacterial infection globally, representing 13.6%, or one in eight, of all global deaths [6]. Investigations conducted in The Gambia using the technique of post-mortem questionnaire, have suggested that about one-half of all neonatal deaths, and a higher proportion of deaths in the second and third months of life, are caused by infections [7,8].

To assist in the design and implementation of effective interventions against IBD, it is important to determine the epidemiology of IBD in Sub-Saharan Africa. However, most health facilities in the region lack the resources to conduct microbiological studies, so data on IBD are sparse. A recent systematic review found few population-based studies reporting the aetiology of IBD in neonates in Sub-Saharan Africa [9]. Previous studies [3,10,11] on the burden and causes of IBD were primarily hospital-based and their findings may not be representative of the population, nor suitable for calculating the true incidence of IBD. Before the introduction of conjugate vaccines against *Haemophilus influenzae* type b (Hib) and pneumococcal conjugate vaccines (PCV), hospital-based studies in Kenya and Mozambique reported that *Escherichia coli*, *Staphylococcus aureus,* and group B *Streptococcus* were the most common pathogens among neonates with sepsis [10,11], while a hospital-based study in the Gambia showed that *S. aureus, Streptococcus pneumoniae,* and *Salmonella spp* were the most important causes of IBD in young Gambian infants [3]. Given the paucity of population-based data on the epidemiology of IBD in young infants in Sub-Saharan Africa, particularly in the era of PCV and Hib vaccination, we evaluated the incidence, aetiology, and clinical outcomes of IBD in young infants in rural Gambia from 2011 to 2017.

## METHODS

### Study design and settings

Between 1 March 2011 and 31 December 2017, we conducted population-based surveillance for IBD among infants aged 0-90 days resident in the Basse Health and Demographic Surveillance System (BHDSS) in Upper River Region, The Gambia (Figure S1 in the **Online Supplementary Document**). Infants admitted to Basse District Hospital and satellite clinics within the surveillance areas had standardised evaluation and investigation using conventional microbiology. Similar surveillance was extended to infants aged 0-90 days, admitted to Bansang Hospital and the satellite clinics in the Fuladu West Health and Demographic Surveillance System (FWHDSS) in Central River Region (Figure S1 in the **Online Supplementary Document**) between 1 September 2011 and 30 September 2014. All infants residing in the surveillance areas were eligible for enrolment. We excluded children who were admitted for observation, trauma or elective surgical procedures and non-residents. Residence was defined as birth to a resident woman. Every resident in the area was assigned a unique 14-digit identifier. The Hib conjugate vaccine was introduced into the Gambian National Programme on Immunization in 1997, and PCV was introduced in 2009.

The BHDSS was established in 2007 and is enumerated every four months. The population in 2017 was estimated to be 184 000 with 5300 annual live births. The BHDSS is served by six satellite clinics and the Basse District Hospital. The FWHDSS was established in 2011, adjoining the BHDSS, and is also enumerated every four months. The population in 2014 was estimated to be 92 464 with 3625 annual live births. The FWHDSS is served by Bansang Hospital and three satellite clinics. Subsistence farming and cattle rearing are the main occupations in both demographic areas.

### Surveillance procedures

Data were collected prospectively at admission and discharge or death for all infants who were admitted to health facilities within the study area. Trained nurses, and/or clinicians, collected data using standardised case report forms and used standardised criteria for the classification of suspected meningitis, sepsis, or pneumonia (Table S1 in the **Online Supplementary Document**). Weight was recorded using a digital scale (TANITA, Arlington Heights, USA) and height using a ShorrBoard® (Weigh and Measure, Olney, USA). Peripheral arterial O_2_ saturation was recorded for all patients using oximetry (Nellcor N-65, Covidien, Colarado). All infants admitted with a possible infectious disease were eligible for blood culture. Blood was collected (1-3 mL) for culture using a sterile technique and inoculated into paediatric BACTEC bottles (Becton Dickinson, Franklin Lakes, NJ, USA) or tryptone soy and brain heart infusion bottles (for a minority of samples collected at night in satellite clinics). Samples collected into BACTEC bottles were incubated in an automated BACTEC 9050 blood culture system (Becton Dickinson, Franklin Lakes, NJ, USA) while samples collected in conventional blood culture bottles were sub-cultured every 24 hours for a maximum of five days. The weight of blood culture bottles was measured before and after sample collection. Patients with suspected meningitis had a lumbar puncture for cerebrospinal fluid (CSF) collection. Chest radiographs were taken on all cases of suspected pneumonia. Lung aspiration was performed in selected cases when the following criteria were met: a large radiographic area of dense, peripheral, pneumonic consolidation; stable respiratory status; and written informed consent provided by the parent or guardian. Pleural fluid was aspirated in selected patients with a large pleural effusion. A finger prick sample was used for rapid measurement of haemoglobin (HemoCue, Ängelholm, Sweden).

### Laboratory methods

BACTEC bottles that signalled positive were sub-cultured onto blood agar, chocolate agar, and McConkey agar. Bottles which failed to signal within five days were considered negative. Isolates were identified using conventional microbiological techniques and biochemical tests (API, Biomerieux). Lung aspirates were transported immediately to the laboratory and inoculated onto agar and examined using Gram’s and Ziehl-Nielson stains. Other sterile site samples were processed using consistent and standardized techniques [12]. Bacterial isolates were defined as contaminants when bacteria generally considered normal skin flora were isolated (coagulase-negative *Staphylococcus*, *Bacillus spp*, *Micrococcus spp*, and *Streptococcus viridans*) [13]. Antimicrobial sensitivity patterns were determined by means of Kirby-Bauer disk diffusion on Mueller-Hinton agar and interpreted according to Clinical Laboratory Standard Institute guidelines [14].

### Case definitions

An episode of IBD was defined as isolation of pathogenic bacteria by culture of blood, CSF, pleural fluid, or lung aspirate [15]. If a patient had bacterial isolates from two or more samples, this was considered as a single episode. If pathogenic bacteria and contaminants were isolated from one sample or from one patient it was considered an IBD episode. Repeated episodes were considered as separate events if the first and subsequent admissions were at least 30 days apart, or if a different bacterial pathogen was isolated on each occasion. We classified IBD as bacteraemia, meningitis, or pneumonia if pathogenic bacteria were isolated from blood, CSF, or lung aspirate and/or pleural fluid samples, respectively.

### Statistical methods

Data were analysed using STATA (version 14, College Station, Texas). Association between categorical variables was tested using Pearson χ^2^ test. The Shapiro-Wilk test was used to test for normality of quantitative variables. We used the *t* test and ANOVA to compare two or more groups of normally distributed data while the Wilcoxon rank-sum test and the Kruskal-Wallis rank test were used when the normality assumption was not met. We considered two-sided *P* < 0.05 as the criterion for statistical significance. The unique 14-digit identifier (HDSS ID) assigned to every patient avoided duplication of patient data in our data set.

We used HDSS enumeration data to estimate the average number of live births each year in each HDSS population and corrected this for incomplete periods of time (January to August 2011 in FWHDSS). Neonatal mortality was estimated as 2% using the HDSS data. To obtain the number of person-days at risk, we multiplied the number of live births by 28 days for infants aged 0-28 days; by 0.98 x 62 days for infants aged 29 to 90 days (accounting for 2% neonatal mortality) and by (28 + 0.98 x 62 days) for infants aged 0-90 days. We calculated person-years at risk by dividing the number of person-days at risk by 365.25. Incidence rates for each year were calculated by dividing the number of IBD cases in the BHDSS and FWHDSS populations in that year by the person-years contributed by each HDSS population. Incidence rates for the whole period were calculated by dividing the number of IBD cases by the sum of person-years contributed by each of the two HDSS populations over the study period. Each incidence rate was multiplied by 1000 to convert the units to per 1000 person-years.

To investigate whether the isolation of *S. aureus* may have been due to sample contamination rather than invasive disease, we examined the relationship between time to culture positivity for 91 *S. aureus* isolates and the child’s clinical outcome using logistic regression. Clinical outcome was defined as poor if outcomes were death, transfer to a higher facility, or persisting disability or length of hospital stay was five days or more. We hypothesised that true pathogens would have shorter time to positivity and lead to poorer outcome than contaminants [16,17]. Furthermore, we assumed that if *S. aureus* isolates sometimes reflected contamination, then poor clinical outcomes would be associated with the time to culture positivity.

## RESULTS

**Figure 1:**
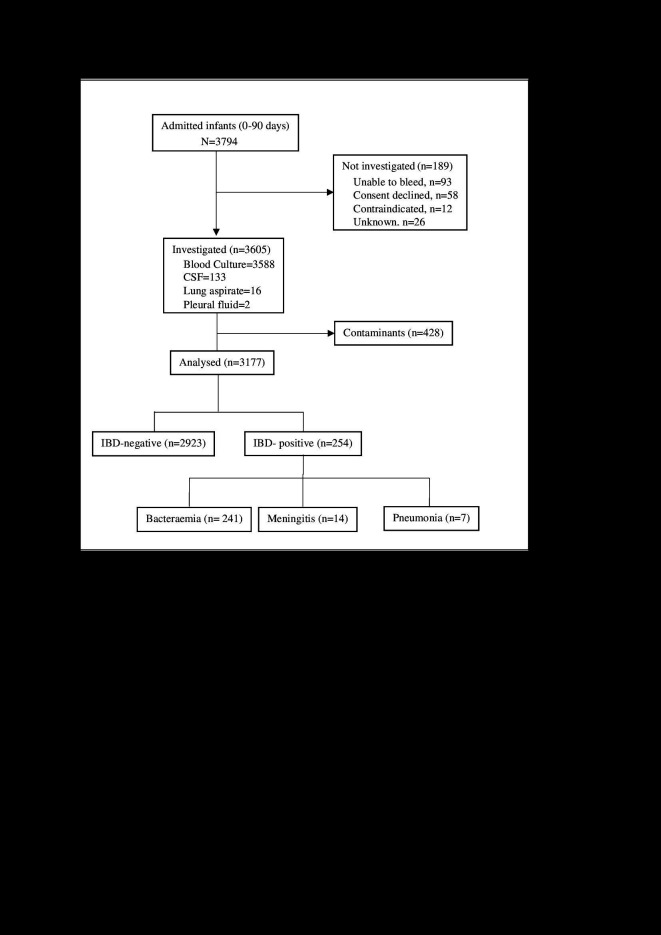
Flowchart of participants included in the study.

During the study period, 3794 infants aged 0-90 days were admitted for acute medical conditions; 3605 (95.0%) had microbiological investigations (3588 blood cultures, 133 CSF cultures, 16 lung aspirates and two pleural fluid cultures). The average volume of blood for culture was 1.26 ml (standard deviation (SD) = 0.68). One hundred eighty-nine admitted infants could not be investigated, mainly due to failed venipuncture or lack of consent (**Figure 1**) and were excluded from analysis. The proportion of deaths among uninvestigated patients was 33/189 (17.5%). These patients had a higher risk of death compared to investigated patients (279/3177 (8.8%), risk ratio (RR) = 1.97; 95% CI = 1.42-2.73; *P* < 0.001). Samples from 428 (11.9%) patients yielded contaminants. There was no difference in mortality between patients with contaminants compared to culture-negative samples (RR = 1.31; 95% CI = 0.96-1.81; *P* = 0.097). Given that contaminants may have masked true infections, we excluded these 428 patients from the analysis. Hence, we included 3177 infants aged 0-90 days in the analysis (**Figure 1**). The characteristics of these patients are described in **Table 1**.

**Table 1:** Characteristics of infants aged 0-90 days admitted with or without invasive bacterial disease in rural Gambia, 2011-2017

Patient characteristic	Patients with IBD, n (%)	Patients without IBD, n (%)	Total, n (%)	*P-*value
Age (in days)				
*≤28*	172 (67.7)	1470 (50.3)	1642 (51.7)	<0.001
*29-90*	82 (32.3)	1453 (49.7)	1535 (48.3)	
Sex				
*Male*	144 (56.7)	1690 (57.8)	1834 (57.7)	0.779
*Female*	110 (43.3)	1233 (42.2)	1343 (42.3)	
Cough	103 (40.6)	1731 (59.3)	1834 (57.7)	<0.001
Difficulty breathing	121 (47.6)	1678 (57.4)	1799 (56.6)	0.008
Diarrhoea	37 (14.6)	310 (10.6)	347 (10.9)	0.040
Unable to feed	101 (39.8)	681 (23.3)	782 (24.6)	<0.001
Convulsion	19 (7.4)	74 (2.5)	93 (2.9)	<0.001
Lethargy	49 (19.3)	252 (8.6)	301 (9.5)	<0.001
Lower chest wall indrawing	75 (29.5)	1325 (45.3)	1400 (44.1)	<0.001
Grunting	28 (11.0)	260 (8.9)	288 (9.1)	0.267
Bulging fontanelle	21 (8.3)	58 (2.0)	79 (2.5)	<0.001
Severe abdominal distension	19 (7.5)	77 (2.6)	96 (3.0)	<0.001
Axillary temperature*> =* 37.5°C	144 (56.7)	1134 (38.8)	1278 (40.2)	<0.001
Tachypnoea*	148 (58.0)	1787 (60.9)	1935 (60.8)	0.640
Tachycardia†	89 (35.0)	1120 (38.3)	1209 (38.1)	0.534
Oxygen saturation <93%	55 (21.6)	476 (16.3)	531 (16.7)	0.076
Haemoglobin <10 g/dl	43 (16.9)	355 (12.2)	398 (12.5)	0.081
Skin infection present	70 (27.6)	465 (15.9)	535 (16.7)	<0.001
Umbilicus infection	32 (12.6)	213 (7.3)	245 (7.7)	0.007
Weight for age z score<-3	48 (18.9)	363 (12.42)	411 (12.9)	0.006
Weight for length z score<-3	68 (26.8)	506 (17.3)	574 (18.1)	<0.001
Mortality	66 (26.0)	213 (7.3)	279 (8.8)	<0.001
Admission days, mean (SD)	4.6 (6.1)	4.3 (3.9)	-	0.672

IBD – invasive bacterial diseases, g – gramme, dl – decilitre, SD – standard devation

*Increased respiratory rate was defined as ≥60 breaths per minute if age <60 days or ≥50 breaths per minute if age ≥60 days.

†Increased pulse rate was defined as >179 beats per minute if age <30 days or >159 beats per minutes if age ≥30 days.

### Characteristics of patients with invasive bacterial diseases

Bacteraemia was identified in 241 (7.6%) of the 3177 infants, seven of whom also had a positive CSF culture. Bacteraemia occurred in 166/1642 (10.1%) neonates compared to 75/1535 (4.9%) infants aged 29-90 days (*P* < 0.001). Fourteen (10.5%) of the 133 patients who had CSF culture had culture-confirmed meningitis, nine of whom were neonates. Seven patients had a positive lung aspirate culture, one of whom also had a positive blood culture. Overall, 254 of 3177 infants (8.0%) included in the analysis had culture confirmed IBD (**Figure 1**). IBD was more common among neonates than infants aged 29-90 days (172/1642 (10.5%) vs. 82/1535 (5.3%); (*P* < 0.001)). Demographic and clinical characteristics of infants with and without IBD are shown in **Table 1**.

### Incidence of IBD and bacteraemia

Using the combined surveillance data (2011-2014 in FWHDSS and 2011-2017 in BHDSS) we found that the incidence of IBD in infants aged 0-90 days was 25 episodes/1000 person-years (95% CI = 22-28). In neonates it was 50 episodes/1000 person-years (95% CI = 43-58) and in infants aged 29-90 days IBD incidence was 12 episodes/1000 person-years (95% CI = 9-15) (Table S2 in the **Online Supplementary Document**). The incidence of bacteraemia was 24 episodes/1000 person-years (95% CI = 21-27) among infants aged 0-90 days. The incidence of bacteraemia was greater in neonates (46 episodes/1000 person-years) than among infants aged 29-90 days (12 episodes/1000 person-years). The incidence of bacteraemia in neonates was 3.1 episodes/1000 live births (Table S2 in the **Online Supplementary Document**).

### Trends in incidence of IBD over time

**Figure 2:**
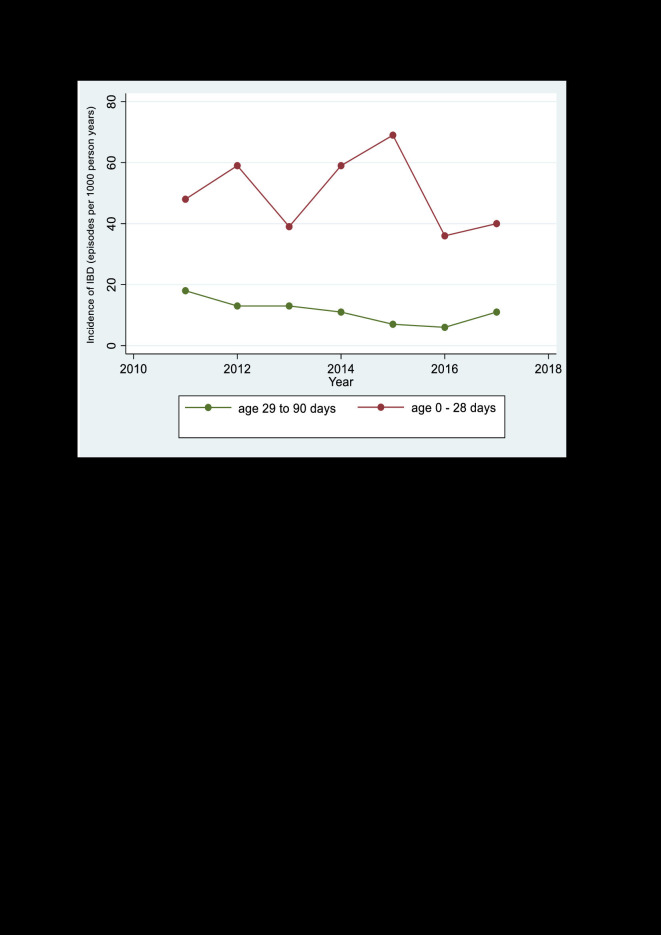
Incidence of invasive bacterial disease in infants aged 0-90 days in the BHDSS rural Gambia, 2011-2017.

Using the BHDSS surveillance data (2011-2017), there was a 39% reduction in the incidence of IBD among infants aged 29 to 90 days, from 18 episodes/1000 person-years in 2011 to 11 episodes/1000 person-years in 2017 (*P*-value for trend = 0.037). There was no significant change in the incidence of IBD in neonates over time (*P*-value for trend = 0.595) (**Figure 2**).

### Bacterial species causing invasive bacterial diseases

**Figure 3:**
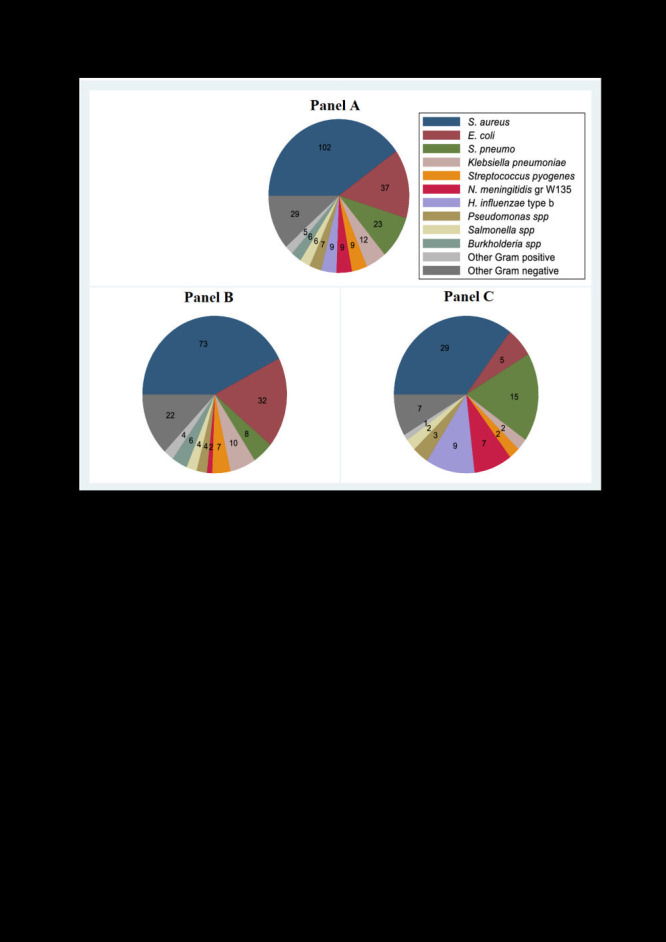
Bacteria isolated in 254 episodes of invasive bacterial disease in young infants in rural Gambia. **Panel A.** Infants aged 0-90 days. **Panel B.** Infants aged 0-28 days. **Panel C.** Infants aged 29-90 days.

The most common bacterial species causing IBD in infants aged 0-90 days, were *S. aureus* (102 episodes; 40.0% of IBD episodes), *E coli* (37 episodes; 14.6% of IBD episodes), *S. pneumoniae* (23 episodes; 9.1% of IBD episodes) and *Klebsiella pneumoniae* (12 episodes; 4.7% of IBD episodes). (**Figure 3**, panel A) These four pathogens accounted for more than two-thirds of the isolates identified. Overall, 55% of IBD episodes were due to Gram-positive bacteria. However, Gram-negative bacteria predominated in the first week of life, causing 61% (47 out of 77 IBD episodes). In the first week of life, there were similar numbers of *E coli* and *S. aureus* IBD episodes (21 and 20 of 77 episodes respectively) followed by *K. pneumoniae*, *S. pneumoniae* and *Burkholderia spp,* with five episodes each. However, *S. aureus* predominated beyond the first week of life, causing 54% (50 out of 92) of IBD episodes in neonates aged eight to 28 days.

*E coli* was significantly more common among neonates with IBD (19.0%) than among infants aged 29-90 days (6.1%; *P* < 0.001). *S. pneumoniae* IBD was less frequent among neonates compared to infants aged 29-90 days (4.7% vs. 18.3%; *P* < 0.001). (**Figure 3**, panel B and panel C) Twelve serotypes (1,2,4,5,7F,10A,12B,13,18A,19A,19F and 35B) were identified among the 23 isolates of *S. pneumoniae* identified. Twelve were serotypes contained in a 13-valent pneumococcal conjugate vaccine: 5 (6 episodes), 19A (2 episodes), and 1 episode each of serotypes 1, 4, 7F and 19F. Eleven were non-vaccine type serotypes: 2 (5 episodes), 13 (2 episodes), and 1 episode each of serotypes 10A, 12B, 13, 18A, and 35B. Only three (1.2% of episodes of IBD) were due to the group B streptococcus (two in neonates). *H. influenzae (*nine Hib and two non-typable) accounted for 11 episodes of IBD in infants aged 29-90 days but zero episodes in neonates. *Neisseria meningitidis* group W was isolated in 1.2% (2/172) of neonates with IBD compared to 8.5% (7/82) of infants aged 29 to 90 days (*P* = 0.005) (**Figure 3**, panel B and panel C).

Bacteria isolated from CSF were *N. meningitidis* group W (n = 4 episodes), *E coli* (n = 3 episodes), *S. pneumoniae* (n = 3 episodes) and one episode each of *Burkholderia spp*, *Chromobacterium spp*, *Enterococcus spp* and *Serratia spp* (**Table 2**). Among seven patients with culture-positive lung and/or pleural samples, *S. aureus* was identified in four, while Hib, *S. pneumoniae* and *H. influenzae* (non-type b) were isolated in one patient each (**Table 2**).

**Table 2:** Prevalence and case-fatality ratio related to bacterial species causing IBD in young infants in rural Gambia

Pathogen	Age					
	**0 to 90 days**		**≤28 days**		**29 to 90 days**	
	No of cases (%)*	**No of deaths (%)†**	No of cases (%)*	**No of deaths (%)†**	No of cases (%)*	**No of deaths (%)†**
**Bacteraemia**						
Gram-positive bacteria						
*Staphylococcus aureus*	99 (41.1)	14 (14.1)	73 (44.0)	11 (15.1)	26 (34.7)	3 (11.5)
*Streptococcus pneumoniae*	22 (9.1)	8 (36.3)	8 (4.8)	3 (37.5)	14 (18.7)	5 (35.7)
*Streptococcus pyogenes*	9 (3.7)	3 (33.3)	7 (4.2)	3 (42.9)	2 (2.7)	0
Others‡	4 (1.7)	0	3 (1.8)	0	1 (1.3)	0
Gram-negative bacteria						
*Eschericia coli*	34 (14.1)	15 (44.1)	29 (17.5)	14 (48.3)	5 (6.3)	1 (20.0)
*Klebsiella pneumoniae*	12 (5.0)	6 (50.0)	10 (6.0)	6 (60.0)	2 (2.5)	0
*Haemophilus influenzae* type *b*	8 (3.3)	0	0	-	8 (10.0)	0
*Neisseria meningitidis* group W135	8 (3.3)	2 (25.0)	2 (1.2)	0	6 (7.5)	2 (33.3)
*Pseudomonas spp*	7 (2.9)	2 (28.6)	4 (2.4)	1 (25.0)	3 (3.8)	1 (33.3)
*Salmonella spp*	6 (2.5)	2 (33.3)	4 (2.4)	1 (25.0)	2 (2.5)	1 (50.0)
*Burkholderia spp*	5 (2.1)	1 (20.0)	5 (3.0)	1 (20.0)	0	0
*Enterobacter spp*	4 (1.7)	3 (75.0)	3 (1.8)	3 (100.0)	1 (1.3)	0
Others§	23 (9.5)	7 (30.4)	18 (10.8)	6 (33.3)	5 (6.3)	1 (20.0)
Total	241	63 (26.1)	166	49 (29.5)	75	14 (18.7)
**Meningitis**						
*Neisseria meningitidis* gr W135	4 (28.6)	1 (25)	0	-	4 (80.0)	(25)
*Eschericia coli*	3 (21.4)	1 (33.3)	3 (33.3)	1 (33.3)	0	-
*Streptococcus pneumoniae*	3 (21.4)	1 (33.3)	2 (22.2)	1 (50.0)	1 (20.0)	0
Others	4 (28.6)	1 (25)	4 (44.4)	1 (25.0)	0	-
Total	14	4 (28.6)	9	3 (33.3)	5	1 (20)
**Pneumonia**						
*Staphylococcus aureus*	4 (57.1)	1 (25)	0	-	4 (57.1)	1 (25)
Others‖	3 (42.9)	0	0	-	3 (42.9)	0
Total	7	1 (14.3)	0	-	7	1 (14.3)

*Percentages refer to the proportion of the isolates in each age group.

†Percentages refer to the proportion of deaths in each age group.

‡Species included: *group B streptococcus* (3 episodes) and *enterococcus* (1 episode)

§Species included: *Acinetobacter spp*, *Morganella morganii*, *Neisseria spp* (3 episodes each), *Coliform*, *Chromobacterium*, *Pasteurella spp*, *Serratia spp* (2 episodes each), *Aeromonas spp*, *Brevundomonas vesicularis*, *Citrobacter freudii*, *Empedobacter bravis*, *Proteus mirabilis* and *Haemophilus influenzae* (non-type b) (1 episode each).

Species included: *Burkholderia spp*, *Enterococcus spp*, *Serratia spp*, *Chromobacterium spp* (1 episode each).

‖Species included: *Haemophilus influenzae type b*, *Streptococcus pneumoniae*, *Haemophilus influenza* (non-type b) (1 episode each).

Fifty-four of the 102 infants (53%) with *S. aureus* IBD had skin and/or umbilical infections while 55 had a temperature ≥37.5°C. The mean time to culture positivity for *S. aureus* isolates was 24.8 hours (SD = 21.4) and 81% alarmed positive within 24 hours. Poor clinical outcome did not vary with the time to *S. aureus* culture positivity (OR = 1.02; *P* = 0.140).

### Case fatality

Sixty-three (26.1%) of the 241 patients with bacteraemia died compared with 216 (7.4%) of 2936 without bacteraemia (*P* < 0.001). The case fatality ratio for bacteraemia among neonates was higher than that in infants aged 29-90 days (29.5% vs. 18.7%; *P* = 0.044). Four of the 14 patients with meningitis (28.6%) died in hospital while only one of seven patients with pneumonia died in hospital (**Table 2**). Overall, the case fatality of IBD was 26% (66 out of 254 episodes). The case-fatality ratio for IBD due to Gram negative bacteria was higher than that due to Gram positive bacteria (40/115 (34.8%) vs. 26/139 (18.7%); *P* = 0.004). Comparing the top four bacteria causing IBD, the case fatality ratios among patients with *K. pneumoniae*, *E.coli*, *S. pneumoniae* and *S. aureus* were 50% (6/12); 43.2% (16/37); 34.8% (8/23) and 14.7% (15/102) respectively.

Using BHDSS and FWHDSS mortality records we calculated that IBD was responsible for a minimum of 7.3% (66 out of 899) of all deaths among infants aged 0-90 days resident in the demographic surveillance areas.

### Antimicrobial susceptibility

The susceptibility of bacterial isolates to commonly used antibiotics in The Gambia is shown in **Table 3** and Figure S2 in the **Online Supplementary Document**. The isolates of *S. aureus* tested were highly susceptible to gentamicin (100%), cefoxitin (100%) and oxacillin (86%) but poorly susceptible to penicillin (7%). *E coli* isolates were highly susceptible to cefotaxime (97%), ciprofloxacin (94%) and gentamicin (90%) but poorly sensitive to cotrimoxazole (31%) and ampicillin (26%). Isolates of *S. pneumoniae* were highly sensitive to penicillin (100%), oxacillin (96%) and ampicillin (86%), while *K. pneumoniae* isolates were highly sensitive to ampicillin (100%), chloramphenicol (91%) and ciprofloxacin (91%) (**Table 3**).

**Table 3:** Antibiotic susceptibility of pathogens associated with invasive bacterial disease among infants aged 0-90 d in rural Gambia, 2011-2017

Pathogens Isolates	Antibiotic susceptibility*										
	Penicillin	Ampicillin	Oxacillin	Cefoxitin	Chloramphenicol	Tetracycline	Cotrimoxazole	Erythromycin	Gentamicin	Cefotaxime	Ciprofloxacin
	n/N (%)	n/N (%)	n/N (%)	n/N (%)	n/N (%)	n/N (%)	n/N (%)	n/N (%)	n/N (%)	n/N (%)	n/N (%)
*Staphylococcus aureus*	6/88 (7)	-	63/73 (86)	23/23 (100)	87/94 (93)	60/88 (68)	56/92 (61)	75/90 (83)	88/88 (100)	-	-
*Eschericia coli*	-	8/31 (26)	-		23/31 (74)	12/21 (57)	10/32 (31)	-	27/30 (90)	30/31 (97)	31/33 (94)
*Streptococcus pneumoniae*	13/13 (100)	12/14 (86)	21/22 (96)		17/24 (71)	5/23 (22)	5/21 (24)	18/21 (86)	-	10/15 (67)	5/13 (39)
*Klebsiella pneumoniae*	-	11/11 (100)	-		10/11 (91)	3/6 (50)	5/10 (50)	-	8/10 (80)	7/10 (70)	10/11 (91)
*Neisseria meningitidis* group W135	0/12 (0)	-	-		11/12 (92)	2/12 (17)	0/12 (0)	-	-	-	10/12 (83)
*Haemophilus influenzae* type b	0/6 (0)	9/9 (100)	-		4/9 (44)	1/8 (13)	1/9 (11)	-	-	-	9/9 (100)
*Streptococcus pyogenes*	7/8 (88)	3/4 (75)	-		6/6 (100)	1/4 (25)	0/4 (0)	-	-	4/4 (100)	1/6 (17)
*Salmonella spp*	-	3/5 (60)	-		3/5 (60)	3/5 (60)	3/5 (60)	-	4/4 (100)	5/5 (100)	4/4 (100)
*Burkholderia spp*	-	-	-		2/4 (50)	-	4/4 (0)	-	-	2/3 (67)	3/4 (75)

*n indicates number of isolates susceptible. N is the total number of isolates tested.

Sixty of all 68 isolates tested (88.2%) were sensitive to a combination of ampicillin and gentamicin (ie, sensitive to at least one of the agents); 100% (86/86) were sensitive to a combination of penicillin and gentamicin; 94.5% (154 out 163) were sensitive to gentamicin only; 47.7% (51 out of 107) were sensitive to ampicillin only while 32.3% (42/130) were sensitive to penicillin only (Figure S2 in the **Online Supplementary Document**).

## DISCUSSION

This study provides population-based surveillance data over a seven-year period on the public health burden of invasive bacterial diseases among infants aged 0-90 days in rural Gambia. We found a high incidence of culture confirmed IBD (25/1000 person-years), particularly among neonates (50/1000 person-years or 3.2 episodes/1000 live births) with a substantial case fatality (26.0%). The predominant bacterial species causing IBD were *S. aureus, E coli, S. pneumoniae* and *K. pneumoniae,* which accounted for more than two-thirds of all IBD cases. Culture confirmed IBD was responsible for a minimum of 7.3% of all deaths among young infants in the study population.

Our observed incidence of IBD in young infants in rural Gambia (4.75/1000 live births) is consistent with that reported previously by a hospital-based study in peri-urban Gambia (4.42/1000 live births) [3]. The incidence that we observed among neonates (3.2 per 1000 livebirths) was lower than previously reported in rural Kenya (5.5 per 1000 livebirths) [10], Nigeria (6.5 cases/1000 livebirths) [18] and Zimbabwe (21 cases per 1000 livebirths) [19]. The lower frequency of IBD observed in our study may be partly explained by the impact of introduction of Hib and pneumococcal conjugate vaccines. However, our observed incidence in neonates is higher than that reported by ANISA, a recent multicenter study in south Asia (1.6 per 1000 livebirths) [20]. This finding could be partly explained by our population-based surveillance in all health facilities in the area which could have included a less severely ill group of patients, and the use of more stringent criteria to classify true pathogens by the ANISA study. We observed a 39% reduction in the incidence of IBD among infants aged 29-90 days, during the period 2011 to 2017 but no significant change was observed among neonates. This reduction may be due to the impact of sustained vaccination with pneumococcal conjugate [21,22] and *Hemophilus influenzae* type b vaccines [23] which are administered to young infants from the second month of life and lack of an approved vaccine against *Staphylococcus aureus*, the most common cause of IBD in neonates.

The aetiology of IBD that we observed is broadly consistent with the findings from a recent systematic review of the aetiology of invasive bacterial infections in neonates in Sub-Saharan Africa [9] and the Global Burden of Disease Study [6]. Gram positive bacteria comprised 55% of the bacteria identified, consistent with the findings from the WHO Young Infant [24] and the Malawian [25] studies. This differs from the recent multicentre study in south Asia [20], and from a study in Madagascar [26], where Gram negative bacteria predominated. *S. aureus* was the primary pathogen in our setting, as in other west and east African studies [9]. Given that 81% of the *S. aureus* isolates alarmed positive within 24 hours and poor clinical outcome did not vary with the time to *S. aureus* culture positivity (OR = 1.02; *P* = 0.140), our findings suggest that *S. aureus* isolates were invasive. The proportion of infection due to *S. pneumoniae* detected was 9.1% (23 out of 241 episodes) compared to 18.9% (10 out of 53 episodes) reported previously in a similar age group in peri-urban Gambia [3]. This suggests a possible decline in prevalence of *S. pneumoniae* post introduction of PCV in Gambia as previously reported [21,22].

Eleven episodes of IBD due to *H. influenzae (*nine Hib and two non-typable) were detected in infants aged 29-90 days. This suggests that Hib transmission continues in The Gambia despite the vaccination programme, as previously reported [23]. We detected only three infections with group B *Streptococcus* (GBS). This finding is consistent with the earlier WHO Young Infant studies [24], but differs from studies in Kenya [10], Malawi [25], and South Africa [27]. A previous Gambian study found that rectovaginal carriage of GBS among women in labour was similar to that reported in developed countries, and significant infant carriage was demonstrated in the same study [28]. This suggests that Gambian infants may be protected from GBS disease, possibly by maternal antibody acquired trans-placentally or through breast milk. Studies from other developing countries in Southern Africa [29,30] and South Asia [20] have shown that in these areas GBS mainly causes early onset neonatal disease. Although 26% (940 out of 3605) of infants investigated in our study were aged less than seven days, the burden of GBS might have been underestimated because of the challenges in collecting samples from severely ill neonates and the possible failure of many early onset disease cases to reach health facilities.

In our study, resistance to recommended empirical antibiotics was generally low, including very few oxacillin- and cefoxitin- (methicillin-) resistant *S. aureus* isolates, compared with the situation in some countries in Sub-Saharan Africa [31,32]. Unlike many other settings in developing countries, no isolates of *S. pneumoniae* were resistant to penicillin, supporting previous finding of a low prevalence of penicillin resistance in The Gambia [33]. We found that ampicillin/gentamicin or penicillin/gentamicin combinations provided significant coverage (88 and 100% respectively) against all bacterial isolates tested, consistent with findings in urban Gambia [34], Kenya [35] and Malawi [25]. This supports the appropriateness of the antibiotics recommended by WHO for the management of potentially serious bacterial infections in young infants [36].

The strengths of our study were that the surveillance was population-based over seven uninterrupted years and that more than 95% of eligible infants had microbiological investigation undertaken using reliable techniques. The number of isolates (264) identified in our study was more than observed in most similar studies in Africa and other resource limited settings [10,20,24]. However, the study also had some limitations. The population was an open cohort, hence our observed incidence of IBD is subject to attrition bias. However, it is consistent with the incidence previously reported in peri-urban Gambia [3]. The incidence of IBD and its case fatality are likely to have been underestimated for several reasons. Culture has limited sensitivity to detect bacterial infection. The isolation rate of blood culture is ~ 10% for infected neonates in most high-income and low-income countries [37]. Small volumes of blood for inoculation and the use of antibiotics prior to investigation may also compromise the sensitivity of blood cultures [38]. Follow-up blood cultures in patients with high fever may increase sensitivity, but only a single blood specimen was collected upon admission in this study. In addition, only admitted infants were investigated and some infants with severe or fatal bacterial infections may never access treatment at a health facility. Although only 5% of admitted infants were not investigated, we found a higher proportion of deaths among them, which could be due to IBD (RR = 1.97; 95% CI = 1.42-2.73). Measuring antibiotic sensitivity using disk diffusion only, without further confirmation may have led to an overestimation of the prevalence of antibiotic resistance [39]. However, our results show a low prevalence of antibiotic resistance in rural Gambia. Although this surveillance was concluded about six years ago, there have not been any significant changes in the Gambian health system that might make the results less relevant to the current situation.

## CONCLUSIONS

The high incidence and mortality associated with invasive bacterial diseases in young Gambian infants underscores the need for maternal and/or neonatal prevention strategies. Low antibiotic resistance in the study setting provides an incentive to limit indiscriminate antibiotic use to prevent emergence of antimicrobial resistance. Overcoming barriers to widespread implementation of existing vaccines and developing new vaccines against the most common pathogens causing IBD should be among top priorities for reducing the high mortality rate in young infants. Additional measures that have been suggested include building of stronger health systems, improved diagnostic and microbiological capacity and implementation of appropriate infection control and antimicrobial stewardship measures [6].

## Additional material


Online Supplementary Document
