# Supplementary material for: Invasive bacterial disease in young infants in rural Gambia: Population-based surveillance
Source: J Glob Health. 2023 Sep 29;13:04106. doi: 10.7189/jogh.13.04106 (PMC10540664; doi:10.7189/jogh.13.04106)
Supplement: Online Supplementary Document [file jogh-13-04106-s001.pdf]

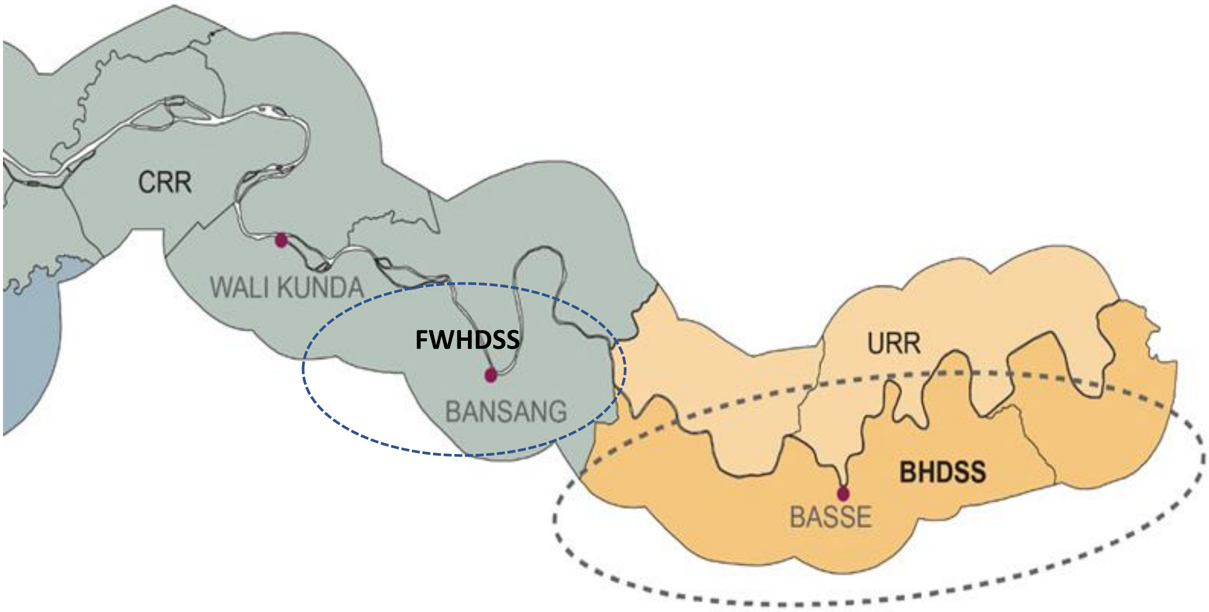

Figure S1: Map of The Gambia showing the Basse and Fuladu West Health and Demographic Surveillance Systems (BHDSS and FWHDSS)

Table S1. Clinical definitions for suspected pneumonia, septicaemia, and meningitis

	**Age <5 years**
**Suspected pneumonia**	Suspected pneumonia is defined if there is a history of cough or difficulty breathing of less than 14 days' duration, accompanied by one or more of:<br>1. Raised respiratory rate for age[*]<br>2. Lower chest wall indrawing, nasal flaring or grunting<br>3. Oxygen saturation less than 92%<br>4. Focal chest signs (dull percussion note, coarse crackles, bronchial breathing)
**Suspected meningitis**	Suspected meningitis will be defined according to clinical judgement and is to be considered if any of the following are present:<br>1. Neck stiffness<br>2. Impaired consciousness[†]<br>3. Prostration[‡]<br>4. History of convulsion<br>5. Bulging fontanelle
**Suspected septicaemia**	Suspected septicaemia will be defined as one or more of:<br>1. Clinician diagnosis of focal sepsis (including but not limited to: septic arthritis, osteomyelitis, endocarditis, peritonitis, liver abscess, soft tissue abscess, cellulitis)<br>2. For a patient admitted, or being admitted, axillary temperature is <36°C or ≥38°C and no obvious cause of fever<br>3. For a patient admitted, or being admitted, the clinical impression is of severe malnutrition

*Raised respiratory rate for age is defined as ≥60 breaths per minute for children less than 2 months of age and ≥50 breaths per minute for children 2 to 11 months of age.
†Impaired consciousness is defined as V, P, or U on the AVPU score, where A is if the patient is alert, V if responsive to verbal stimulus, P if responsive to pain stimulus, and U if unresponsive.
‡Prostration is defined as inability to drink or breast feed.

Table S2: Incidence of invasive bacterial disease and bacteraemia in infants aged 0-90 in rural Gambia, 2011 -2017*

Age group (days)	No. episodes/no. person-years at risk	Incidence (95% CI), per 1,000 person-years	Incidence rate ratio (95% CI)	*P*-value
		**Invasive bacterial disease**		
≤28	172/3494	49.2 (42.1 – 57.1)	4.11 (3.14 – 5.42)	<0.0001
29 to 90	82/6849	12.0 (9.5 – 14.9)	Referent	
0 to 90	254/10343	24.6 (21.6 – 27.8)	-	
		**Bacteraemia**		
≤28	166/3494	47.5 (40.6 – 55.3)	4.34 (3.28 – 5.78)	
29 to 90	75/6849	11.0 (8.6 -13.7)	Referent	
0 to 90	241/10343	23.3 (20.5 – 26.4)	-	<0.0001
		**Meningitis**		
≤28	9/3494	2.6 (1.2 - 4.9)	3.53 (1.06 – 13.4)	
29 to 90	5/6849	0.7 (0.2 – 1.7)	Referent	0.0236
0 to 90	14/10343	1.4 (0.7 – 2.3)	-	
		**Pneumonia**		
≤28	0	-	-	
29 - 90	7/6849	1.0 (0.4 – 2.1)	-	
0 - 90	7/10343	0.6 (0.2 – 1.3)	-	

* Surveillance data were from March 2011 to December 2017 in BHDSS and from September 2011 to September 2014 in FWHDSS

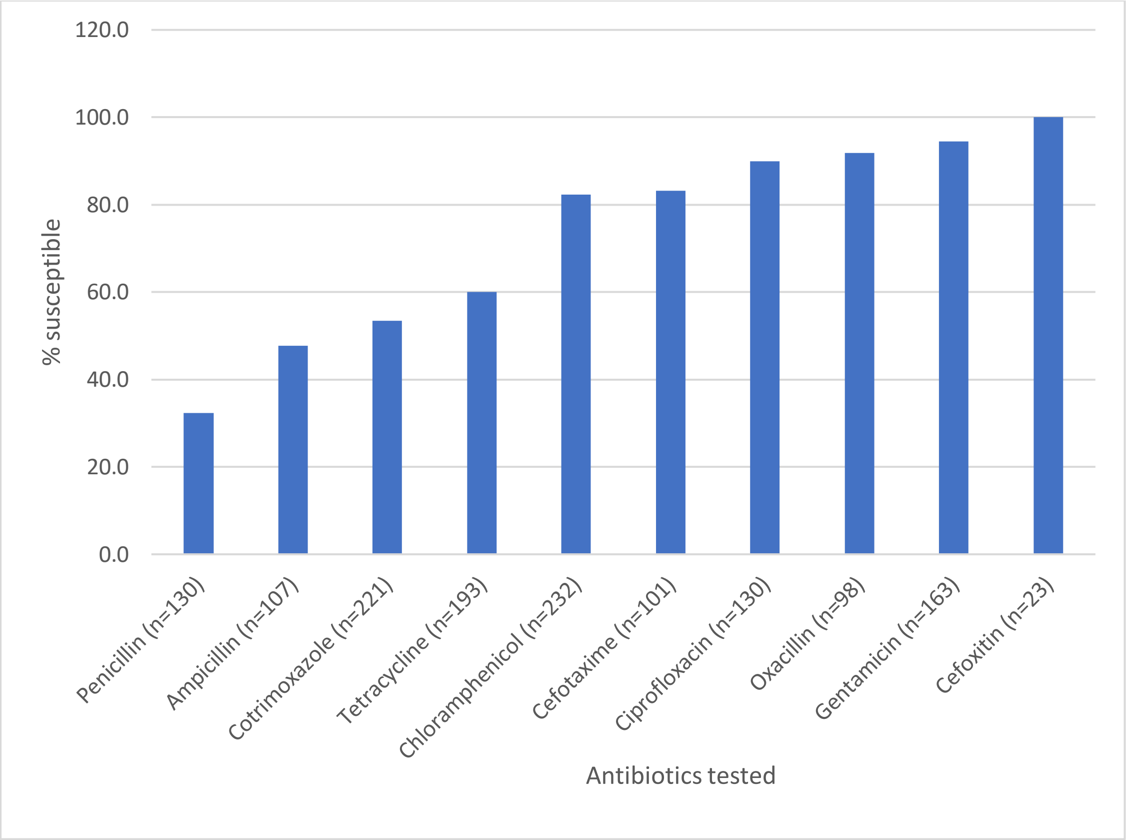

Figure S2: Antibiotic susceptibility of invasive bacterial isolates in infants aged 0-90 days in rural Gambia, 2011-2017
